# Preimplantation Genetic Screening: An Effective Testing for Infertile and Repeated Miscarriage Patients?

**DOI:** 10.1155/2010/120130

**Published:** 2010-07-01

**Authors:** Ning Wang, Ying-Ming Zheng, Lei Li, Fan Jin

**Affiliations:** Department of Reproductive Endocrinology, Women's Hospital, School of Medicine, Zhejiang University, Hangzhou 310006, China

## Abstract

Aneuploidy in pregnancy is known to increase with advanced maternal age (AMA) and associate with repeated implantation failure (RIF), and repeated miscarriage (RM). Preimplantation genetic screening (PGS) has been introduced into clinical practice, screening, and eliminating aneuploidy embryos, which can improve the chance of conceptions for infertility cases with poor prognosis. These patients are a good target group to assess the possible benefit of aneuploidy screening. Although practiced widely throughout the world, there still exist some doubts about the efficacy of this technique. Recent randomized trials were not as desirable as we expected, suggesting that PGS needs to be reconsidered. The aim of this review is to discuss the efficacy of PGS.

## 1. Introduction

Preimplantation genetic screening (PGS) has been used more than 10 years for selecting genetically normal embryos giving the highest potential for preimplantation genetic diagnosis (PGD). PGS usually involves the aspiration of the first polar body from oocyte before fertilization or one or two cells from a 5- to 8-cell embryo 3 days after insemination. Fluorescence in situ hybridization (FISH), is often performed, using probes for a specific number of chromosomes most commonly involved in aneuploidy. The presence or absence of a normal pair of chromosomes can be identified visually by color, so we can eliminate the abnormal embryos and select normal embryos for transfer [1]. Thus, choosing embryos selected by PGS with normal chromosomes should increase implantation rate and live-birth rate and reduce miscarriages. The indications of PGS include advanced maternal age (AMA), repeated implantation failure (RIF), repeated miscarriage (RM), and severe male-factor infertility [2].

Currently, there have been a great many studies into in vitro fertilization (IVF) or intracytoplasmic sperm injection (ICSI) with and without PGS. Several of them found that selecting embryos with normal chromosomes had a significant impact on the implantation rate compared with the controls [3–6]. PGS has been advocated as a valuable tool for embryo screening. In recent years, its trends become controversial after the report published by Mastenbroek et al. [7]. They found that PGS reduced the rates of pregnancies and live births after IVF in women of AMA [7]. Actually Mastenbroek was not the only one who claimed that PGS might not be as beneficial as expected. Conclusions drawn from other studies in AMA patients after PGS showed that PGS did not significantly improve implantation rate and pregnancy rate, on the contrary it worsened the outcome [8, 9].

In this paper, we will acknowledge the importance of aneuploidy screening and review the findings of currently published studies of PGS, in order to discuss the efficacy of this technique.

## 2. Indications of PGS in AMA, RM, RIFand Severe Male-Factor Infertility

Aneuploidies, that is, deviations from the regular number of chromosomes, are predominantly the result of maldistributions of chromosomes during meiosis. Aneuploidy rates in oocytes and embryos are known to increase with maternal age [10]. In a 40-year-old woman, an estimated 50% to 70% of the mature oocytes are affected by a chromosomal abnormality [11, 12]. In a series of 6733 oocytes obtained during 1297 IVF cycles from patients of AMA (mean 38.5 years) [13], 3509 (52%) were aneuploidy, on the basis of FISH analysis using specific probes for chromosomes 13, 16, 18, 21, and 22. It is well known that the age-related increase in aneuploidy rate is correlated with a reduced implantation and a higher abortion rate. Most evidence collected so far suggests that failed implantation due to embryo aneuploidy rather than failed conception is the primary cause responsible for low human fertility [14]. To date, these patients revealed an aneuploidy rate of over 50%, suggesting the practical relevance of PGS to women of advanced reproductive age. Screening for aneuploidy in preimplantation embryos may help select the best embryo to transfer and may open the way to significant improvements in live-birth rates from IVF/ICSI, especially relevant for more effective single embryo transfer [15]. AMA patients, here defined as ≥35 years, are a good target group to assess the possible benefit of aneuploidy screening. 

RM is defined when two or more consecutive spontaneous abortions occur, which affects 1% of couples trying to conceive [16]. The number of miscarriages stands out as a predictor of the chromosome abnormality rate,whichis directly proportional to the number of miscarriages. A study of 108 couples with history of repeated abortions found that chromosome abnormalities were found in 5% of the couples with two abortions, in 10.3% with three abortions, and in 14.3% with four or more abortions [17]. The most common anomaly observed in abortus is aneuploidy, and reported aneuploidy rate could reach to 34–66% [18, 19]. This result suggested that aneuploidy was a common cause of RM, and led to the proposal that PGS may be beneficial in these patients.

RIF can be defined as the failure of a couple to conceive after the transfer of 10 or more good-quality embryos, or after three IVF cycles [20]. Although multiple aetiologies, such as disturbed endometrial receptivity, uterine pathology, and an inadequate transfer technique, have been proposed, increased incidence of numerical chromosomal abnormalities is obviously the most common cause [21]. It has been reported that the rate of chromosome abnormalities in the embryos from RIF patients is almost twice as much as that in the controls (67.4% versus 36.3%) [22]. Significantly higher incidence of complex chromosome abnormalities (which involves three or more chromosomes) was also found in RIF [23]. The generation of aneuploidy embryos was considered as a possible causative factor in RIF [24], and it is suggested that PGS may improve the outcome in these patients.

Infertile couples due to severe male factor can be treated with ICSI. In order to generate normally fertilized oocytes after ICSI, a spermatozoon containing a functional genome and centriole is required [25]. Current study in cases of macrocephalic spermatozoa demonstrated an increased incidence of chromosomal abnormalities, and the majority of the abnormalities were aneuploidy [26, 27]. Due to the high incidence of aneuploidy these patients might benefit from PGS owing to its effect of eliminating chromosomally abnormal embryos.

## 3. Studies with Beneficial Outcome of PGS

### 3.1. PGS in AMA

An early study published by Gianaroli et al. [3] on 157 cycles (73 for PGS group and 84 controls) with AMA using FISH in analysis of chromosomes X, Y, 13, 14, 15, 16, 18, 21, and 22 in a blastomere biopsied from day 3 embryos showed that 64% of embryos presented with chromosomal abnormalities. 22 cycles in the study group had clinical pregnancies versus 25 cycles in the control group, whereas in the study group, the mean number of embryos transferred per patient was significantly lower (2.2 ± 0.9 versus 3.2 ± 0.9), and the implantation rate was higher in comparison with the control group (25.8% versus 14.3%; *P* < .01). Concomitantly, the implantation rate per pregnant patient was superior in the study group compared with the controls (57.9% versus 38.5%; *P* < .05). More interestingly, these patients were arbitrarily divided into three classes of age: 36–37 years, 38–39 years, and ≥40 years; the pregnancy and implantation rates characterized in the control group revealed a significant decrease when patients aged ≥38 years. Conversely, in the study group, the percentages of pregnancy and implantation did not differ among the three classes of age, and the implantation rate observed in the oldest categories (≥38 years) was significantly higher after aneuploidy screening than the controls. 

Verlinsky et al. [28] performed a study of polar body diagnosis (PBD) with IVF cycles from patients of AMA. 5590 oocytes were obtained from 917 cycles and tested by polar body sampling and FISH analysis using specific probes for chromosomes 13, 16, 18, 21, and 22, this resulted in 22.2% clinical pregnancies and 140 healthy children born. It seems that polar body testing provides an approach for improving pregnancy rate in IVF patients of AMA. But no control group was presented in this report. Another study of women ageing 35 to 39 years with two or more previous IVF/ICSI treatment trials showed that a higher implantation rate was achieved in the PBD group (17.5% versus 11.8%) [29]. These results suggested that an indication-based use of PBD could certainly provide benefits in older patients.

Some articles showed that aneuploidy screening in preimplantation embryos can also reduce embryo loss, increasing ongoing pregnancies and delivery rates. Munné et al. [5] designed a multicentre IVF study to compare controls and a test group that underwent aneuploidy screening, obtaining a significant improvement in the number of spontaneous abortions and ongoing pregnancies. Similar beneficial effects have been reported by other studies. Staessen et al. [8] observed a trend toward a subsequent higher ongoing implantation per transferred embryo rate in tested group (16.5% versus 10.4%; *P* = .06). In the recent study published in 2009 by Schoolcraft et al. [30], 62 infertile AMA couples undergoing fertility treatment were assigned to the PGS and control group. Results showed that the implantation rates, the number of oocytes, oocyte maturity, and fertilization rate were similar between the two groups. Nevertheless, the authors noted that the spontaneous abortion rate was lower for the test group (25.9% versus 32.26% in the control group), resulting in an observed increase in delivery rate for the test group (78% versus 67.74%). In addition, Hardarson et al. [9] found significantly more good morphological quality embryos (GQEs) in the PGS group on day 3 compared with those found in the control group.

### 3.2. PGS in RM, RIF, and Severe Male-Factor Infertility

The randomized, prospective study including 19 couples with recurrent pregnancy loss (11 for PGS and 8 controls) by Werlin et al. suggested an improved outcome after performing PGS [31]. Pregnancy rate was 63.6% in study group and 37.5% in controls. In another study performed by Munné et al. [18], the rate of spontaneous abortions in RM subjects undergoing PGS was compared with their own a priori expectations. After PGS, miscarriage rate was reduced from previous 90% (expected 29%) to 23% in the women at age <35 years, and from 86% (expected 44.5%) to 12% in the women at age ≥35 years. Similar results were also reported by a multicenter retrospective controlled study [32], which showed that the spontaneous abortion rate in the PGS group was 14.1% for women ages 35–40 and 22.2% for over 40, compared to 19.4% (*P* < .03) and 40.6% in the non-PGS group (*P* < .001). 

Improved outcomes in RIF were achieved with the selection of chromosomally normal embryos. In a study with 57 RIF cycles by Pehlivan et al. [22], a pregnancy rate of 34.0% and an implantation rate of 19.8% was observed in the PGS group. Recent data reported that, in women with unexplained RIF [33], two consecutive PGS cycles showing euploidy embryo(s) were strongly associated with high ongoing pregnancy (40%) and implantation (18%) rates. Conversely, the patients with no euploid embryos in a PGS cycle were highly unlikely to achieve an ongoing pregnancy in subsequent cycles.

Kahraman et al. compared the implantation and ongoing-pregnancy rates of PGS cycles with non-PGS cycles in cases with predominantly macrocephalic spermatozoa and absolute teratozoospermia [34]. A statistically higher implantation rate as well as a significantly reduced missed abortion rate were found in PGS group (25.0% and 14.3%) compared with non-PGS group (12.3% and 46.7%).

## 4. Studies without Beneficial Outcome of PGS

### 4.1. PGS in AMA

In the study by Staessen et al. [8] used FISH for the chromosomes X, Y, 13, 16, 18, 21, and 22 in AMA couples with a control group without PGS. In the 400 (200 for PGS and 200 controls) couples were allocated to the trial, ICSI was used to fertilize the oocytes, and two blastomeres per embryo were removed on day 3 after injection and transferred on day 5. In this study, the implantation rates were not significantly different between the two groups (17.1% in the test group versus 11.5% in the control group). But the cycles that had embryos transferred were significantly lower in test group (81 cycles versus 121; *P* < .001), and 38 couples in the test group had no genetically normal embryos to transfer. Less than expected success of PGS was attributed to a higher number of embryos transferred in the control group (2.8 versus 2.0) and the possible adverse effect of double-blastomere biopsy [35]. The same group, comparing single-cell versus two-cell biopsy, demonstrated a detrimental effect of two-cell biopsy; they suggested that, if one-cell biopsy had been used in their study, implantation rates may have improved.

Mastenbroek et al. [7] designed a multicenter, randomized, double-blind, controlled trial. 408 women of AMA underwent 836 cycles of IVF, of which 206 women with 434 cycles were assigned to PGS and 202 women with 402 cycles to the control group. The ongoing-pregnancy rate was significantly lower in the women assigned to PGS (52 of 206 women, 25%) than in those not assigned to PGS (74 of 202 women, 37%). The women assigned to PGS also had a significantly lower live-birth rate (24% versus 35%) and reduced implantation rate of (11.7% versus 14.7%) compared with those in the control group. The study was criticized mainly for inappropriate patient selection, inadequate probe selection, possible biopsy-induced embryo damage, a low average number of embryos biopsied, and a high rate of undiagnosed embryos [36, 37].

In the Hardarson et al. study [9], 56 and 53 patients with age ≥38 years were randomly assigned to the PGS and control groups, respectively. Fertilization was performed by IVF or ICSI following standard techniques and FISH analyzed by probes chromosomes X, Y, 13, 16, 18, 21, and 22 in PGS group. Of the analyzed embryos (302 embryos), only 32.4% (98 of 302) had normal chromosome content and 70 of 98 normal embryos were transferred. The number of patients who had embryos transferred was 45 (80.3%) in PGS group and 53 (100%) in control group (*P* = .001). The clinical pregnancy rate/randomized patient in the PGS group was 8.9% compared with 24.5% in the control group (*P* = .039). No significant differences were found in the implantation rates (11.4% versus 18.9%) or live-birth rate (5.4% versus 18.9%) per randomized patient between the PGS group and the control group. As shown in these randomized trials, no improvement in efficacy was observed.

### 4.2. PGS in RM, RIF, and Severe Male-Factor Infertility

Platteau et al. designed prospective cohort PGS study in women with recurrent idiopathic miscarriages [19]. The pregnancy results in the older group (≥37 years) were disappointing, with an implantation rate of 2.77% and an ongoing-pregnancy rate of 2.94%. The probable cause for this poor result was that these older women had significantly more chromosomally abnormal embryos than patients <37 years (66.95% versus 43.85%). 

As to the RIF, a prospectively randomized controlled trial of PGS in IVF/ICSI patients with recurrent failed implantation compared with conventional assisted reproduction treatment procedures was carried out by Blockeel et al. [38]. A total of 139 patients underwent ovarian stimulation, and PGS was performed in 72 patients. No benefit to their implantation and clinical pregnancy rates was found. The implantation rate was 21.4% in the study group and 25.3% in the control group. Moreover, the clinical pregnancy rate was much lower in the study group (25.0% versus 40.3%). 

Although severe male-factor infertility is one of the PGS indications that have been put forward, current reports of PGS in severe male-factor infertility are rare. There is a lack of scientific evidence to prove whether PGS is effective in these patients.

## 5. Reasons for Lack of Benefit in PGS

Technical reasons for lack of benefit in PGS include both biopsy damage to the remaining embryo that reduces its developmental potential and limitations of current FISH technology that allows only a few chromosomes to be seen. As a result, it is inevitable that some other abnormal chromosomes will escape from detection [39]. Moreover, FISH could be misdiagnosed by the probability of hybridization failure and the possibility that the fluorescent signals of two chromosomes overlap each other. The testing of all chromosomes would probably further increase observed aneuploidy rates [40]. Mosaicism, a difference of the chromosomal constitution among individual cells in an embryo, is another possible reason for confusion. A single blastomere that had been biopsied might thus be classified as abnormal, whereas the remaining blastomeres in the embryo are normal. Thus, the test results from the biopsied cell may not be an accurate indication of the embryo's genetic status [41]. Besides technical limitations and mosaicism, contamination and laboratory mistakes can also result in inaccurate diagnoses. For example, DNA from sources other than the biopsied cell may be read as part of the genetic analysis, mixup, or mislabeling of a sample or embryo from clinic or laboratory mistakes in handling samples or embryos. All of these can lead to inaccurate results.

## 6. Possible Future Trends in PGS

### 6.1. Comparative Genomic Hybridization (CGH)

Performed on a single cell basis, CGH enables the assessment of all the chromosomes by comparing the studied DNA with a normal sample. In brief, normal DNA samples are labelled with red and test DNA with green fluorochromes, and then applied to a slide where hybridization occurs for 48–72 h [42]. The advantage of CGH over the conventional FISH is that the copy number of all chromosomes can be determined. CGH can provide a genome-wide profile without any prior information of the chromosomal aberration [40]. 

Fragouli et al. [43] collected 270 oocytes from the 16 female patients (average age 38.4 years) and 168 embryos were fertilized on day 3 (average 12 embryos per patient, range 6–18). Of the 168 embryos, 78 (46.4%) were cultured further to the blastocyst stage and underwent trophectoderm biopsy with CGH screening. Their data displayed high implantation and pregnancy rates for the patients with RIF who have received blastocyst analysis [43]. CGH yielded results for 73 of the 78 blastocysts, leading to a diagnostic efficacy of 94%. Of these, 40 were classified as euploidy and 24 were transferred in 13 patients, leading to nine ongoing pregnancies from 13 completed cycles (69.2%) and the implantation rate was 58.3% (14/24 ETs). The limitations of CGH are that it is time-consuming and labour intensive. The long period required for hybridization (5 days) has limited the widespread clinical implementation of this technique, as it is necessary to freeze all the embryos after the biopsy. In addition, the survival rate of the thawed embryos was relatively poor (46% did not survive the thawing process). More recently, the development of highly efficient techniques has greatly reduced fears concerning the impact of cryopreservation on embryo viability. Array CGH is one of the newest technologies developed for the detection of a chromosomal imbalance; it is able to analyze the very limited amount of genetic material in a single cell and takes less time [42]. Accuracy microarray platforms also can offer the advantage of embryo fingerprinting and the potential for combined aneuploidy and single-gene disorder diagnosis [44]. The first report to show a pregnancy after PGS using array CGH technology by Hellani et al. obtained a high pregnancy rate; six out of a total eight patients had embryos for transfer with five out of those six showing positive pregnancy tests [45]. The result was encouraging and further studies on array CGH with larger sample sizes will be required before it is suitable for clinical application. However, some disadvantages need to be addressed before array-CGH is suitable for clinical services. First of all, the accuracy needs further evaluation. Array sometimes gave incorrect results for chromosomes 2, 4, 9, 11, 17 and 22 [46]. Partial aneuploidy and imbalance of chromosome segments are not currently detected. Besides, the present array CGH protocol is expensive and it doesn't seem to fit easily into all clinical PGS services. This requires us to find new ways to reduce costs and bring the advantages to more patients.

### 6.2. Blastocyst Biopsy

Blastocyst biopsy or trophectoderm biopsy is an emerging technique for performing PGS. It shows several advantages over traditional day-3 biopsy [46]. One of them is more cells can be biopsied for genetic testing without damaging the inner cell mass. Biopsy at this stage has little, if any, impact on the further development of the blastocysts. The data from McArthur et al. demonstrates high blastocyst survival rates with excellent implantation rates and low rates of twinning or miscarriage [47]. Recently, a study involving 399 egg retrievals and 1879 embryo biopsies for patients undergoing PGD to avoid a serious monogenic disease or an unbalanced chromosomal translocation has been published. The implantation rates per embryo transferred were 43.4% if biopsied at the blastocyst stage and 25.6% if biopsied at the cleavage stage (*P* < .01), with ongoing or live-birth pregnancy rates per egg retrieval at 34.2% (average transfer number 1.1) for blastocyst biopsies and 25.5% (transfer number 1.6) for cleavage stage biopsies (*P* < .05). The results mean that taking the biopsy later in embryo development conferred considerable efficacy through not testing embryos whose development was compromised [48]. Nevertheless, more data is still needed to confirm these promising results.

## 7. Conclusions

 In conclusion, the efficacy of PGS is still controversial. According to the studies, there is still insufficient evidence to support a beneficial effect of PGS in AMA women. The use of PGS applied for RM, RIF and severe male factor infertility needs more scientific data from clinical trials. The routine use of PGS to avert the birth of an aneuploidy infant is still in question. Application of micro-CGH and blastocyst biopsy might be new approaches for improvement of the efficacy of PGS. Furthermore, the cost-effectiveness of PGS for the IVF patients should be considered.
